# Gynecomastia Surgery: Liposuction Alone versus Liposuction with Endoscope-Assisted Glandular Excision—A Comparative Study

**DOI:** 10.1055/s-0045-1802327

**Published:** 2025-01-23

**Authors:** Sheikh Sarfraz Ali, Imran Ahmed, Mohammed Fahud Khurram, Noha Rehman, Rupraj Abhishek

**Affiliations:** 1 Department of Plastic Surgery, Jawaharlal Nehru Medical College and Hospital, Aligarh Muslim University, Aligarh, Uttar Pradesh, India

**Keywords:** gynecomastia, liposuction, aesthetic outcomes, peri-areolar glandular excision, patient satisfaction

## Abstract

**Background:**

Gynecomastia, affecting around 30% of young males, has seen evolving surgical treatments, transitioning from traditional excision methods to contemporary techniques like liposuction. Emotional distress persists when glandular tissue is inadequately addressed, prompting exploration of combined liposuction and glandular excision procedures.

**Materials and Methods:**

Patients undergoing gynecomastia surgery over a period of 2 years were assessed, considering their demographics, medical history, and gynecomastia grade. Surgical procedures involved liposuction alone or with glandular excision. Endoscopy was used to assess the presence of fibroglandular tissue and the need for glandular excision. Postoperative assessments, clinical photography, and patient questionnaires spanned a 6-month follow-up.

**Results:**

Thirty-two breasts (17 in liposuction alone and 15 in liposuction with glandular excision groups) were included. Liposuction alone led to bruising and two hematomas, and “puffy nipples” necessitating one redo surgery. Glandular excision resulted in four cases of crater deformity and one case each of superficial skin necrosis, hematoma, and seroma. Cosmetic evaluations showed similar outcomes, with the liposuction alone group having higher redo surgery rates.

**Conclusion:**

This study finds that combining liposuction with glandular excision delivers comparable cosmetic results to liposuction alone for gynecomastia. Despite added complexity, the combined approach proves effective and helps in decision-making, emphasizing the need for tailored techniques and ongoing research to optimize treatment strategies.

## Introduction

Gynecomastia refers to the benign growth of the male breast. It has a prevalence of around 30% in young males. 1 It happens because of an imbalance in the ratio of estrogen to testosterone. The most effective approach to address physiological gynecomastia is through reassurance. 2 The underlying disease must be addressed to treat pathological gynecomastia. Over time, the surgical treatment of gynecomastia has evolved, advancing from the excision of breast tissue with or without skin removal using lunate inframammary incisions to the intra-areolar incision and finally to liposuction. 3

Gynecomastia patients also experience sadness, poor body image, and low self-esteem. 4 Any procedure that triggers memories of prior disease is certain to have an adverse effect on the patient's mental health and may not fully address the ailment. Therefore, the major goal of gynecomastia surgery is scarless surgery. This issue is effectively solved with liposuction, which leaves barely noticeable scars. Although adipose tissue can be removed by liposuction, the glandular tissue that is frequently left behind results in an unattractive end result, continuing to give the patient emotional distress and embarrassment. It is believed that treating grade IIb and III gynecomastia with liposuction alone does not solve the issue of skin excess. 3 The fundamental tenet of gynecomastia surgery would be violated if the extra skin was removed because it would leave unsightly scars. In more severe cases of gynecomastia, liposuction combined with excision of the glands by a small incision is an effective treatment option. 5 6 7 8 9 In this study, we assess the clinical and aesthetic results of gynecomastia patients after vacuum-assisted liposuction (VAL) and peri-areolar glandular excision. We contrast the outcomes with liposuction alone as well.

## Materials and Methods

After obtaining the required informed consent and ethical approval, patients who underwent gynecomastia surgery in the department of plastic surgery between February 2022 and February 2024 were included in the study. A thorough history was collected, including drug use and jaundice. The grade of gynecomastia was evaluated through examination. Abdominal examination, assessment of secondary sexual characteristics, and testicular examination to rule out any cancers were also performed. Blood tests were performed as part of the pre-anesthesia workup to rule out liver diseases and to check the hormone levels if needed. Breast ultrasound imaging was done. Clinical photography was done under proper lighting against blue/green background in standard positions.

### Operative Technique

Preoperative markings were done in the standing position on the day of surgery. All the surgeries were performed under general anesthesia.

### Liposuction

**Fig. 1:**
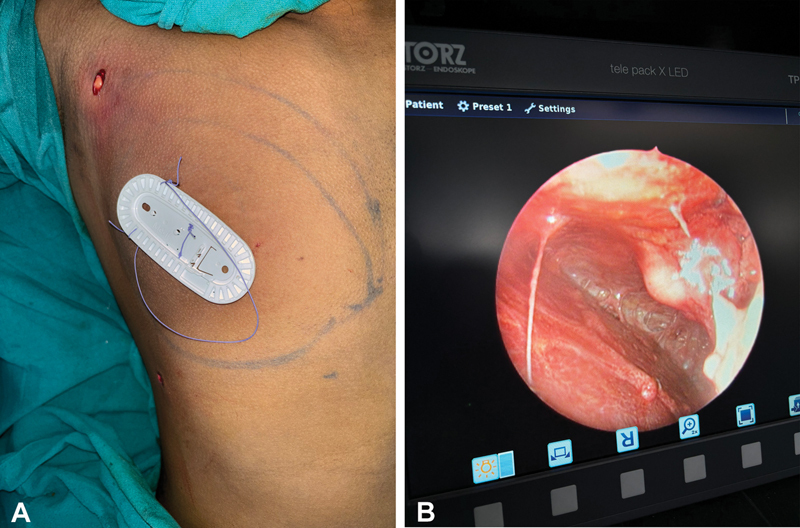
( **A** ) Suture packet holding technique to lift the skin and subcutaneous tissue off the pectoralis muscle. ( **B** ) Endoscopic view of the excess glandular tissue below the nipple areola complex.

The surgery was performed with the patient in the supine position with both arms in 90 degrees of abduction under general anesthesia. Following meticulous painting and draping, a 4-mm stab incision was placed in the anterior axillary line within the anterior axillary fold. Tumescent solution infiltration into the breast tissue and its surrounding area was executed using a blunt-tipped 4-mm infiltrating cannula (Klein's infiltration cannula). A second stab incision was made in the anterior axillary line at the level of the inframammary fold after 7 minutes allowing for the tumescent to start its action. Employing a 3.5-mm blunt-tip cannula (Coleman cannula), VAL was meticulously performed, adhering to established surgical protocols. For fine-tuning and precision fat removal toward the end of the procedure, a 3-mm spiral cannula was employed. This meticulous approach ensured the achievement of the desired chest contour. An endoscope (Karl Storz Endoskope TP100) was introduced via the liposuction port. Suture packet technique as shown in Fig. 1A was used to lift the skin and subcutaneous tissue off the pectoralis muscle and fascia to create space for visualization. If extra-glandular tissue was visualized ( Fig. 1B ), patients were planned for glandular excision. In patients in whom there was no excess glandular tissue, the endoscope was removed while simultaneously placing drains via the inferior port site. Compression dressing was done.

### Glandular Excision

The patient was prepared to undergo glandular excision if extra-glandular tissue was found after liposuction by endoscopic evaluation, as discussed earlier. An inferior peri-areolar incision was made that ran from the 3 o'clock position to the 9 o'clock position. Metzenbaum scissors were used to cut the glandular tissue free from the anterior skin attachments. Using Allis forceps, the gland was grabbed, extracted through the incision, and excised. To avoid adhesions, care was taken not to breach the pectoralis fascia. Once hemostasis was attained, suction drains were cautiously introduced through the inferior liposuction incision to prevent complications such as hematoma or seroma. Monocryl sutures were used to close the incision in a subcuticular manner. A compression dressing was applied to optimize postoperative recovery.

In the instances where excess skin did not necessitate excision, the approach focused on glandular tissue removal. Excised glandular tissue was sent for histopathological examination.

Drains were removed after surgery when the output was less than 30 mL for 24 hours. The dressing was changed on the fifth postoperative day, following which patients were required to wear custom-made compression garments with a simple dressing over port sites. Patients were followed up on days 3, 5, and 14 and then monthly for 6 months, and clinical photography was done. The clinical photographs were assessed by two plastic surgeons for cosmetic evaluation using a 5-point Likert scale in terms of symmetry, nipple areola complex (NAC), scar, and flatness (1: very dissatisfied; 2: dissatisfied; 3 - neither; 4: satisfied; and 5: very satisfied). Patients were required to fill a breast evaluation questionnaire at the 6-month follow-up visit. The questionnaire consisted of three parts with satisfaction assessed by a 5-point Likert scale (1: very dissatisfied; 2: dissatisfied; 3: neither; 4: satisfied; and 5: very satisfied).

## Results

A total of 32 breasts were included in this study, with 17 undergoing liposuction with glandular excision and 15 undergoing liposuction alone. Six patients had bilateral gynecomastia, while eight patients had asymmetrical breasts. In the liposuction with glandular excision group, three patients (four breasts) presented with Simon's grade IIb and III gynecomastia. No statistically significant demographic differences were noted between the two groups ( Table 1 ). Most cases were idiopathic, with three patients reporting a history of steroid intake. Ultrasonography confirmed increased adipose and glandular tissue in all patients.

**Table 1:** Demographic and clinical data of patients undergoing liposuction alone and liposuction with glandular excision

	Liposuction alone	Liposuction + glandular excision
No. of breasts ( *n* )	17	15
Mean age (y)	22.6	23.8
Body mass index (BMI)	26.2	25.8
**Grade (** ***n*** **)**		
I IIa IIb III	8 6 3 0	5 6 3 1
Total operative time (min)	46	65
Fat volume aspirated (mL)	325	310
Mean duration of hospital stay (d)	3.5	4.2
Mean follow-up (mo)	7.4	6.8

Endoscopic visualization added 5 to 10 minutes, and glandular excision required an additional 10 to 15 minutes in the combined procedure group. Drains were removed by postoperative day 3, except in one patient. All patients were discharged between postoperative days 3 and 5.

In the liposuction-only group, skin bruising occurred in three patients (four breasts), resolving spontaneously ( Table 2 ). Two patients developed hematomas, which were managed conservatively. There were no immediate postoperative contour deformities. All but one patient expressed satisfaction with the surgery. One patient required a redo surgery at the 3-month follow-up due to persistent “puffy nipples.”

**Table 2:** Complications following gynecomastia surgery

Complications	Liposuction	Liposuction + glandular excision
Bruising	4	1
Hematoma	2	1
Seroma	0	1
Hypo- or hyperesthesia	1	5
Wound dehiscence	0	0
Infection	0	0
Irregularities	2 (puffy nipples)	4 (crater deformity)
NAC necrosis (partial or total)	0	0
Superficial skin necrosis	0	1
Redo surgery	2	0

Abbreviation: NAC, nipple areola complex.

In the liposuction plus glandular excision group, three patients (four breasts) developed a crater deformity below the NAC, likely from over-resection. This was corrected with fine-tuning liposuction using smaller-diameter spiral cannulas. Residual crater deformities resolved over time with tissue remodeling. One patient experienced superficial skin necrosis of the NAC, which healed with dressings. Hematoma and seroma occurred in one breast each, both resolving without intervention. One patient developed a hypopigmented scar.

At the 6-month follow-up, two patients with redundant skin achieved a normal chest appearance without requiring additional intervention. Patients undergoing liposuction with glandular excision had comparable cosmetic outcomes to those managed with liposuction alone. However, the liposuction-only group had a higher rate of redo surgeries.

**Fig. 2:**
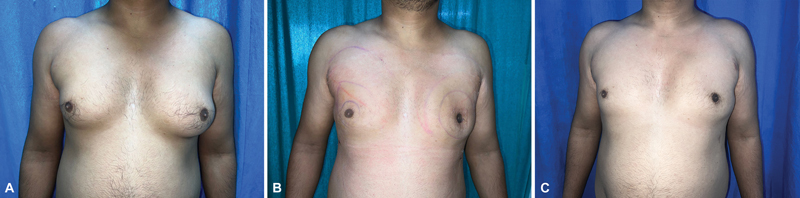
( **A** ) Right breast Simon's grade IIb and left breast grade III gynecomastia. ( **B** ) Day 3 postoperative image with right-sided liposuction and left-sided liposuction with glandular excision. ( **C** ) The 6-month follow-up image.

**Fig. 3:**
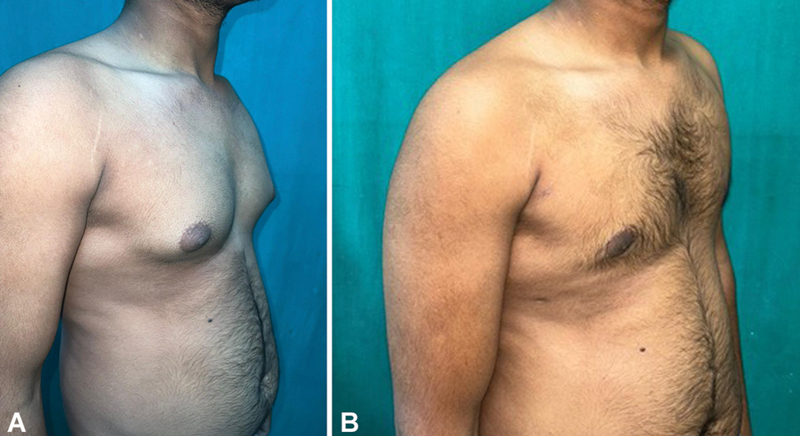
( **A** ) Preoperative image of bilateral Simon's grade IIb gynecomastia. ( **B** ) The 6-month follow-up image of bilateral Simon's grade IIb gynecomastia.

**Fig. 4:**
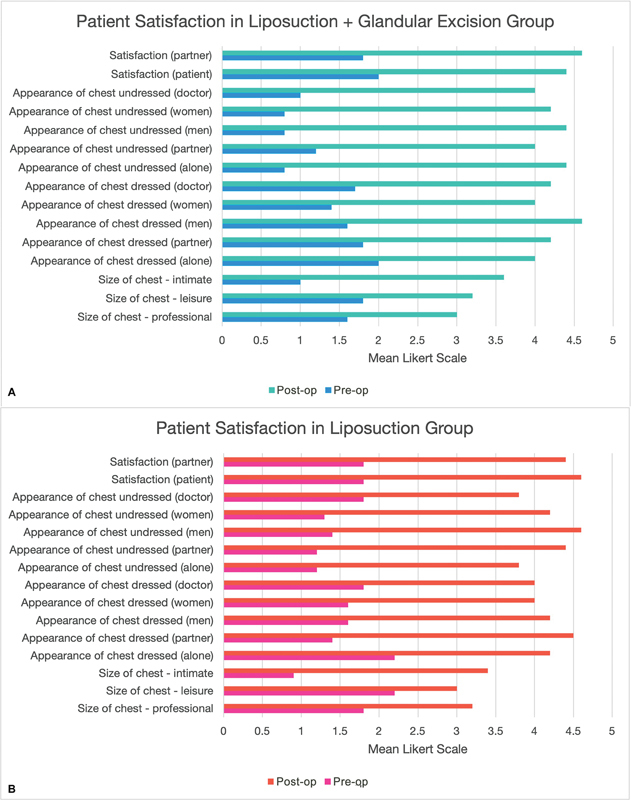
( **A** ) Patient satisfaction (liposuction alone group). ( **B** ) Patient satisfaction (liposuction and glandular excision group).

**Fig. 5:**
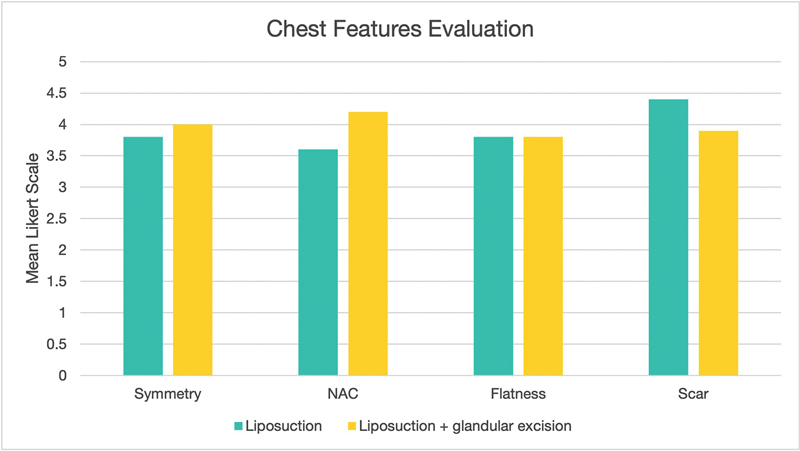
Postoperative satisfaction with chest-specific features. NAC, nipple areola complex.

Fig. 2 illustrates the preoperative, postoperative 3-day (with left-sided crater deformity), and 6-month follow-up images of a patient treated with right-sided liposuction and left-sided liposuction plus glandular excision. Fig. 3 displays the preoperative and 6-month follow-up images of a patient with bilateral gynecomastia treated with bilateral liposuction and glandular excision. Patient satisfaction and chest feature satisfaction are depicted in Figs. 4 and 5 , respectively.

## Discussion

Contemporary challenges in treating gynecomastia include concerns like “puffy nipples,” recurrence, and compromised aesthetic outcomes due to incomplete tissue removal. 10 Liposuction enables minimally invasive tissue removal, yielding enhanced quality of life and satisfaction. 9 Open excision, per Innocenti et al, allows direct hemostasis control and histopathological analysis, vital for detecting rare breast cancer cases. 11

Contrary to studies like Arvind et al involving excess skin removal, our study focused on glandular excision and demonstrated improved cosmetic outcomes with innovative surgical techniques. 12 Addressing concerns about risks with cutting cannulas, our approach, inspired by Tarallo et al, used pretunneling and suction before excision for contouring and ease of glandular removal. 13 The peri-areolar scar, as seen in Arvind et al, was well accepted, and our low complication rate, including hematoma management, highlights the safety of our approach. 12

Combining ultrasonic-assisted liposculpture (UAL) with peri-areolar gland excision (without skin resection) ensures safe and effective outcomes, particularly for grade III gynecomastia. 14 Standard techniques often integrate UAL with conventional liposuction and partial gland resection, minimizing morbidity and maximizing aesthetic results. 15

Abdelrahman et al's study, using a specialized fat disruptor cannula, highlighted glandular tissue breakdown without UAL or gland excision, showing promise for low-resource settings. 16 Our study adopted a similar method due to UAL unavailability. While Abdelrahman et al excluded cases with skin excess, our patients with skin excess did not require excision, achieving comparable outcomes.

Tarallo et al emphasized sequential liposculpture postexcision to enhance skin redraping and minimize irregularities. 13 Asal et al recommended leaving approximately 5 mm of retro-areolar disk tissue to prevent retraction, highlighting the importance of careful glandular excision. 8 Abdali et al compared liposuction with/without skin incision, showing higher satisfaction for the latter, consistent with our findings of high satisfaction and low complications. 7

Preoperative counseling, as suggested by Ridha et al 17 and Hasanyn and Said, 14 is vital for managing expectations. Consistent with Prasetyono et al, our results reinforce the positive impact of liposuction and glandular excision on satisfaction and quality of life. 9 Financial barriers, noted by Alnaim et al, were reflected in our diverse patient cohort, with 31.1% being students with no income. 18

Innocenti et al highlighted complications like hematomas, seromas, and pathological scars, commonly associated with surgical excision. 11 Our findings align with those of Alnaim et al, with reduced complication rates when combining excision and aspiration techniques. 18

Endoscopic evaluation in our study provided a more objective assessment of glandular tissue, preventing unnecessary excisions. Despite added procedural time and complexity, it proved valuable for fibrous tissue visualization. Offering endoscopy without financial burden to low-income patients was an institutional advantage.

Limitations include the small sample size, lack of power-assisted liposuction, and restricted bed availability, necessitating routine use of drains. Targeting specific grades, we plan further exploration of endoscopic evaluation in larger cohorts.

## Conclusion

This study highlights that the cosmetic outcomes of liposuction with glandular excision are comparable to liposuction alone in the management of gynecomastia. Despite the heightened procedural complexity and potential complications associated with the combined approach, it demonstrates effectiveness. The importance of tailoring surgical techniques to individual patient needs remains paramount, underscoring the continual necessity for research to further refine and optimize gynecomastia treatment strategies.
